# 

**Published:** 1906-08-15

**Authors:** 


### ANNOUNCEMENTS.

Michigan Dental Association.—The fiftieth annual meeting of this society was held July 9th, 10th and 11th, in Detroit, at the Detroit College of Medicine buildings. This was the semi-centennial convention and an effort was made to celebrate the aniversary in a suitable manner. As the Association was organized in Detroit fifty years ago, it was thought proper to hold this meeting in that city. An effort was made to get an attendance of the older members and to make the recital of historical events a prominent feature of the program. Through an unfortunate incident this feature was not carried out as planned. It was found quite difficult to rehearse the actual transactions of the early meetings as the record books of the times have been lost and there was no one present whose memory could reproduce actual events as they sequentially happened. Drs. A. F. Metcalf and J. A. Watting presented important contributions which will be published giving the main incidents connected with the Association's work. A committee of five was appointed to endeavor to restore the old records as completely as may be practicable. Dr. W. H. Jackson, of Ann Arbor, is Chairman of this committee and will be glad to learn of any event connected with the association's history during the past fifty years. The program was one of unusual interest and value. Eight papers were read and discussed, and nearly forty clinics were given. The papers were good and were well discussed. The paper which created the most interest was presented by Dr. D. D. Smith, of Philadelphia, on "The Necessity of Reform in Dental Teaching." Dr. Smith is the advocate of dental prophylaxis as the greatest factor in saving the teeth from destruction, and he takes high ground which not all teachers are yet ready to accept. The usual results of too many clinics and a large crowd which wanted to see everything that is new, were experienced. The clinic mania seems to be growing faster than we are developing methods for utilizing it. We must have fewer clinicians and a more orderly method of presenting them than at present if we are to get adequate results.

The Detroit dentists, as usual, showed their liberal hospitality by taking the whole convention with the wives and sweethearts for a boat ride to the "*Flats.*" It was a beautiful day and the ride and entertainment on the boat was all that could be desired.

The following officers were elected for the ensuing year: President, Dr. A. L. Le Gro, of Three Rivers; Vice-president, Dr. E. B. Spalding, of Detroit; Secretary, L. N. Hogarth, of Detroit; Treasurer, Dr. J. Ward House, of Grand Rapids; Legal Trustee, Dr. Geo. Zederbaum, of Charlotte. The next meeting is to be held at Saginaw.

Death of Dr. Stainton.—Charles Wesley Stainton, of Buffalo, N. Y., died at his home in Buffalo, N. Y., June 6, 1906, in the sixty-eighth year of his age. He began the study of dentistry in 1864 in Danville, N. Y., with Drs. Quigby and Daboll, and in 1866 he began practicing for himself. He afterwards attented the Pennsylvania Dental College from which he graduated in 1872. He began practice in Buffalo in 1872, where he has been in practice ever since. He has been a strong factor in all local societies of his district, the New York State Society, and the National Dental Association. He has been treasurer of the New York State Dental Society for many years. He was a frequent contributor to dental literature and society discussions and clinics. He was a man of force and strong personality and yet a man of a courteous and sympathetic nature. He was a true professional man and did much for the profession in its formative period. He will be missed and mourned by a large circle of friends and acquaintances.

Will Celebrate the Fiftieth Anniversary.—The St. Louis Dental Society (the oldest dental society in continuous existence) is contemplating the celebration of its fiftieth anniversary during the month of November. A special committee is at work on the program and hope to have it ready for the September Journals.

